# FGF21 does not require interscapular brown adipose tissue and improves liver metabolic profile in animal models of obesity and insulin-resistance

**DOI:** 10.1038/srep11382

**Published:** 2015-07-08

**Authors:** Barbara Bernardo, Min Lu, Gautam Bandyopadhyay, Pingping Li, Yingjiang Zhou, Jie Huang, Nancy Levin, Eva M. Tomas, Roberto A. Calle, Derek M. Erion, Timothy P. Rolph, Martin Brenner, Saswata Talukdar

**Affiliations:** 1 Cardiovascular Metabolic and Endocrine Diseases (CVMED) Pfizer, Inc. 610 Main Street, Cambridge, MA 02139, USA; 2 Department of Medicine, University of California, San Diego, 9500 Gilman Drive, La Jolla, CA 92093, USA; 3 CovX Research, Pfizer WRD, USA

## Abstract

FGF21 is a key metabolic regulator modulating physiological processes and its pharmacological administration improves metabolic profile in preclinical species and humans. We used native-FGF21 and a long-acting FGF21 (PF-05231023), to determine the contribution of liver and brown adipose tissue (BAT) towards metabolic improvements in Zucker rats and DIO mice (DIOs). FGF21 improved glucose tolerance and liver insulin sensitivity in Zuckers without affecting BW and improved liver function by decreased lipogenesis, increased fatty acid oxidation and improved insulin signaling. Through detailed lipidomic analyses of liver metabolites in DIOs, we demonstrate that FGF21 favorably alters liver metabolism. We observed a dose-dependent increase of [^18^F]-FDG-glucose uptake in interscapular BAT (iBAT) of DIOs upon FGF21 administration. Upon excision of iBAT (X-BAT) and administration of FGF21 to mice housed at 80 °F or 72 °F, the favorable effects of FGF21 on BW and glucose excursion were fully retained in both sham and X-BAT animals. Taken together, we demonstrate the liver as an organ that integrates the actions of FGF21 and provide metabolic benefits of FGF21 in Zucker rats and DIOs. Finally, our data demonstrates iBAT does not play a role in mediating favorable metabolic effects of FGF21 administration in DIOs housed at 80 °F or 72 °F.

Originally identified as an endocrine factor that increases glucose uptake in fat cells^1^, the physiological role of FGF21 was later shown to modulate metabolic processes required for the body’s adaptation to starvation^2^. In human adipocytes, FGF21 mediated glucose uptake is synergistic with insulin^1^,^3^. Pharmacological administration of FGF21 serves as a potent antidiabetic agent in preclinical species demonstrated by improved insulin sensitivity and glycemic control, dyslipidemia, energy expenditure and weight loss in obese mice^4^,^5^,^6^. Moreover, in type 2 diabetic subjects, FGF21 decreased BW and improved lipid profile^7^. The metabolic benefits of pharmacologically administered FGF21 are conferred through its action on the receptor FGFR1c and cofactor beta klotho (KLB)^8^,^9^. Importantly, both FGFR1c and KLB are required for FGF21 action, since the loss of one abolishes the metabolic effect of FGF21^9^,^10^. Given that both FGFR1c and KLB are required for FGF21 action^9^,^10^,^11^, and since expression of FGFR1c is lacking in liver^12^, FGF21 likely does not have a direct effect on the rodent liver^13^,^14^. Adipose tissue and the CNS are key target tissues of FGF21 action and emerging evidence suggests FGF21-mediated increase in adiponectin mediates glycemic control on hepatic glucose production and insulin responsiveness^15^, whereas, weight loss is driven to a large extent through action of FGF21 on the CNS^16^.

In mice, FGF21 causes browning of white adipose cells^17^ and administration of FGF21 increases energy expenditure (EE)^4^,^6^,^18^. In addition, the leptin pathway likely plays a role in FGF21-mediated weight loss since ob/ob and db/db mice have little to no weight loss upon FGF21 administration^14^,^18^,^19^,^20^. A previous report demonstrated BAT-independent actions of FGF21 administration on energy expenditure upon excision of iBAT^21^, however, this study^21^ did not report data on sham-surgery animals administered vehicle or FGF21 in parallel, to allow this conclusion to be drawn.

In this study, we used native FGF21 and a previously reported long-acting FGF21 molecule PF-05231023^18^,^22^ in Zucker fatty rats (LepR^fa^) and DIO mice and measured metabolic endpoints. FGF21 administration in Zucker rats improved glucose homeostasis in the absence of weight loss or changes in food intake. The improved glycemic profile resulted from improved liver metabolism and insulin signaling. Detailed lipidomic analyses from livers of DIO mice revealed robust effects of PF-05231023 on fatty acid oxidation, glycolysis, bile acid metabolism, and glycogen metabolism. Finally, we report a dose-dependent increase of [^18^F]-FDG uptake in BAT of DIO mice upon FGF21 treatment implying improved BAT activity; however, upon iBAT excision and administration of FGF21 in animals housed at 80 °F (which is close to thermoneutrality) or 72 °F (which represents ambient temperature), we demonstrate all metabolic effects of FGF21 are retained, suggesting BAT is not required for FGF21 action in DIO mice. Our study demonstrates the pharmacologic effects of FGF21 in Zucker rats, confirming dependence on the leptin pathway for weight loss and leptin-independent pathways for glycemic control and for the first time that the liver integrates beneficial effects of FGF21 in this species. Finally, our study demonstrates that although BAT has been demonstrated as a target tissue for FGF21 action, it is not required for its metabolic effects in mice dosed for two weeks.

## Results

### Administration of native FGF21 and PF-05231023 improves glucose tolerance and liver insulin sensitivity in Zucker rats

**Figure 1:**
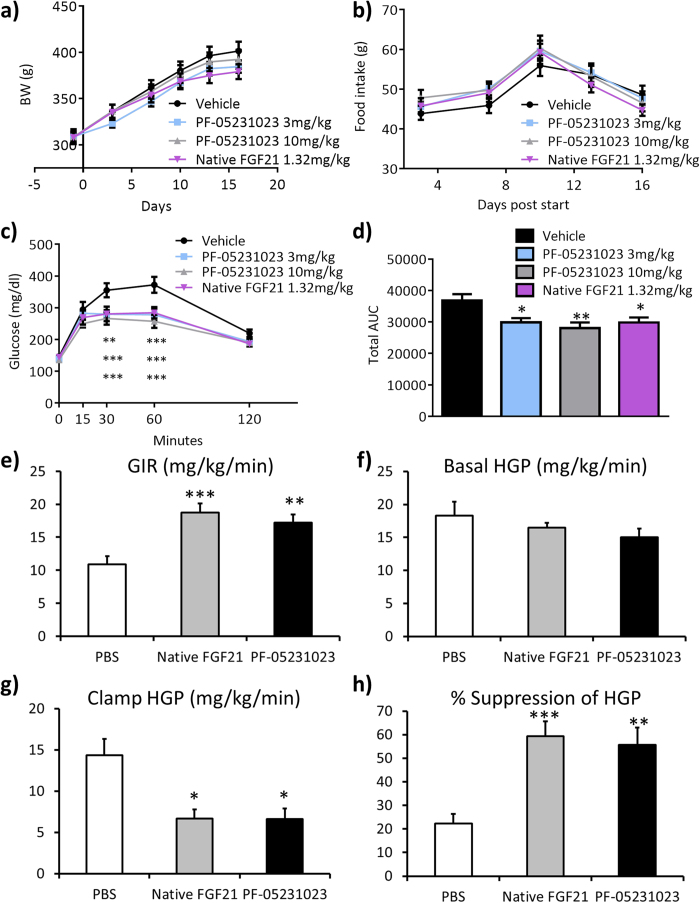
FGF21 improves glucose tolerance in Zucker rats. Eight week old Zucker rats were administered vehicle, 1.32 mg/kg/day native FGF21, or 3 mg/kg and 10 mg/kg PF-05231023 subcutaneously twice a week for two weeks. **a**) body weight (BW), **b**) food intake, **c**) oral glucose tolerance test (OGTT), **d**) area under the curve (AUC), **e**) glucose infusion rate (GIR), **f**) basal hepatic glucose production (HGP), **g**) clamp hepatic glucose production, **h**) suppression of hepatic glucose production. n = 8–10 animals in each group. All data represented as Mean ± SEM. *p < 0.05, **p < 0.01, ***p < 0.001 by one way Anova and Dunnett’s posthoc test.

Eight week old Zucker rats were administered a daily subcutaneous (SC) injection of vehicle, 1.32 mg/kg native FGF21, or a SC injection of 3 mg/kg or 10 mg/kg PF-05231023 twice a week for two weeks. There was no change of BW observed in animals injected with either native, or PF-05231023 (Fig. 1a), and food intake was not changed in the treated groups compared to control (Fig. 1b). An oral glucose tolerance test (OGTT) was performed in these animals on day 14 and both native and PF-05231023 caused a significant decrease of glucose excursion during the OGTT compared to control, although the glucose lowering effect of PF-05231023 maxed out at 3 mg/kg (Fig. 1c). Total area under the curve (AUC) during the OGTT was similarly decreased in all treatment groups (Fig. 1d).

Since OGTTs showed improved glucose tolerance in Zucker rats administered native- and PF-05231023, we wanted to determine the tissue-specific effects of these compounds and performed hyperinsulinemic-euglycemic clamp studies administered vehicle, 1.32 mg/kg/day of native- and 10 mg/kg PF-05231023 for two weeks. The glucose infusion rate (GIR) required to maintain euglycemia was significantly higher in native FGF21 and PF-05231023-treated Zucker rats compared to vehicle and there was no difference in GIR between native FGF21 and PF-05231023 (Fig. 1e). Consistent with lack of effect of native FGF21 and PF-05231023 on fasting glucose after two weeks of treatment, with no difference in basal hepatic glucose production (HGP) between groups (Fig. 1f). However, both native FGF21 and PF-05231023 caused a significant decrease in HGP under clamp conditions, but there was no difference between treatments (Fig. 1g). Consequently, both native FGF21 and PF-05231023 showed a significant increase in suppression of HGP (Fig. 1h). There was no change in clamp GDR (Supplementary Fig. S1a) suggesting liver as the key tissue mediating these effects of FGF21 on glycemia.

### FGF21 improves glucose homeostasis in Zucker rats by improving liver function

**Figure 2:**
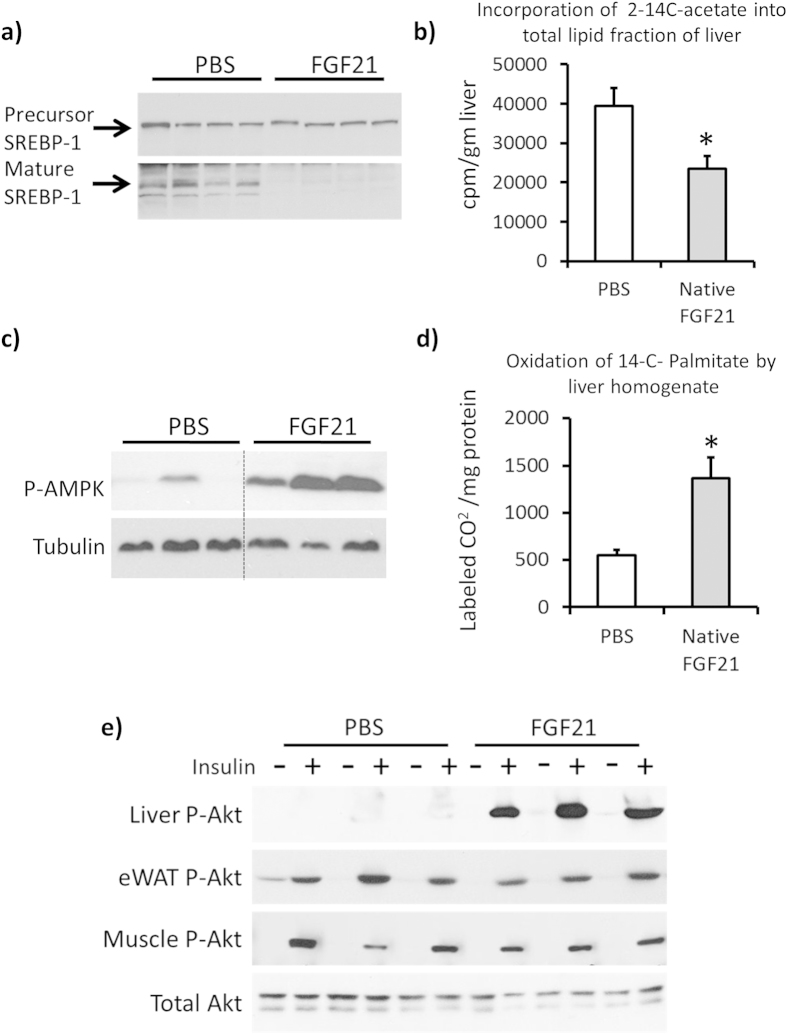
FGF21 improves hepatic metabolic profile in Zucker rats. Livers from Zucker rats administered 1.32 mg/kg/day native FGF21 for two weeks were harvested. **a**) representative western blots for precursor and mature SREBP1, **b**) incorporation of 2-14C-acetate in *ex vivo* livers, n = 6/group, **c**) western blots for P-AMPK (Thr 172) and Tubulin, **d**) 14C incorporation into CO_2_ from *ex vivo* liver slices, n = 6/group. **e**) Acute insulin response in Zuckers administered 1.32 mg/kg/day native FGF21 for two weeks. Western blots for P-Akt and total Akt from the indicated tissues. In Figures a and c, each lane represents a different animal. All gels reported in **a**), **c**) and **e**) were run under the same experimental conditions on the same gels respectively for each of these proteins. In **a**) each lane represents liver lysates from a separate animal, 4 administered PBS and 4 administered FGF21. All samples were run in the order shown in the figure. In **c**) data is shown for 3 animals administered PBS and 3 animals administered FGF21. Each protein was run on its own gel. Dotted line indicates where blots were cropped. In **e**) data is shown for 3 animals administered PBS and 3 administered FGF21. All samples were run in the order shown in the gels. Representative total Akt blot is from liver. Each protein was run on its own gel. Data represented as Mean ± SEM. * p < 0.05 by paired t-test.

Since clamp data showed FGF21 administration improved hepatic insulin sensitivity, we wanted to determine the mechanism(s) by which FGF21 mediates this effect. After two weeks of treatment, native FGF21 decreased the mature active form of SREBP1 in the liver without changing the precursor form (Fig. 2a). Consistent with this finding, hepatic lipogenesis, measured *ex vivo* by incorporation of 2-^14^C-acetate into total lipid fraction of livers from treated animals was significantly decreased in FGF21-treated Zucker livers compared to control (Fig. 2b). Native FGF21 treatment increased hepatic P-AMPK compared to control (Fig. 2c), and subsequently, *ex vivo* hepatic lipid oxidation measured by detecting labeled CO_2_ from ^14^C-palmitate was significantly increased in FGF21 treated animals compared to controls (Fig. 2d). Since EGP was decreased by FGF21 under clamp conditions, we performed acute insulin administration in fasting animals treated with vehicle and native FGF21 for two weeks. On the day of performing the experiment, the treated animals received one final dose of native FGF21 followed by the assessment of insulin signaling 2 hours later. Consistent with data obtained from clamp studies, P-Akt was significantly increased in liver upon acute insulin administration in FGF21 treated animals compared to controls, however no changes in P-Akt was observed in adipose tissue or muscle (Fig. 2e). These data support the findings that liver is the primary tissue responsible for FGF21 action in Zucker rats.

### PF-05231023 improves liver metabolism in DIO mice

We conducted thorough lipidomic and metabolomic analyses to determine the effects of pharmacological FGF21 administration in DIO mice administered vehicle or PF-05231023 for two weeks. Essential fatty acids such as linoleate and all long-chain fatty acids (LCFAs) species other than myristoleate were reduced in the livers of the mice treated with FGF21 (Table 1). In contrast, carnitine, choline, and choline phosphate along with ketone bodies such as beta-hydroxybutyrate levels were elevated in the livers (Table 1). Bile acid metabolite concentrations such as cholate and cholate-derived conjugation products were markedly reduced in the FGF21 livers (Table 1). Lastly, many key gluconeogenic amino acids such as glutamate were reduced in the FGF21-treated mice (Table 1). Overall, this profile was consistent with increased β-oxidation, and reduced insulin-stimulated hepatic glucose production.

### FGF21 administration improves brown adipose tissue function

**Figure 3:**
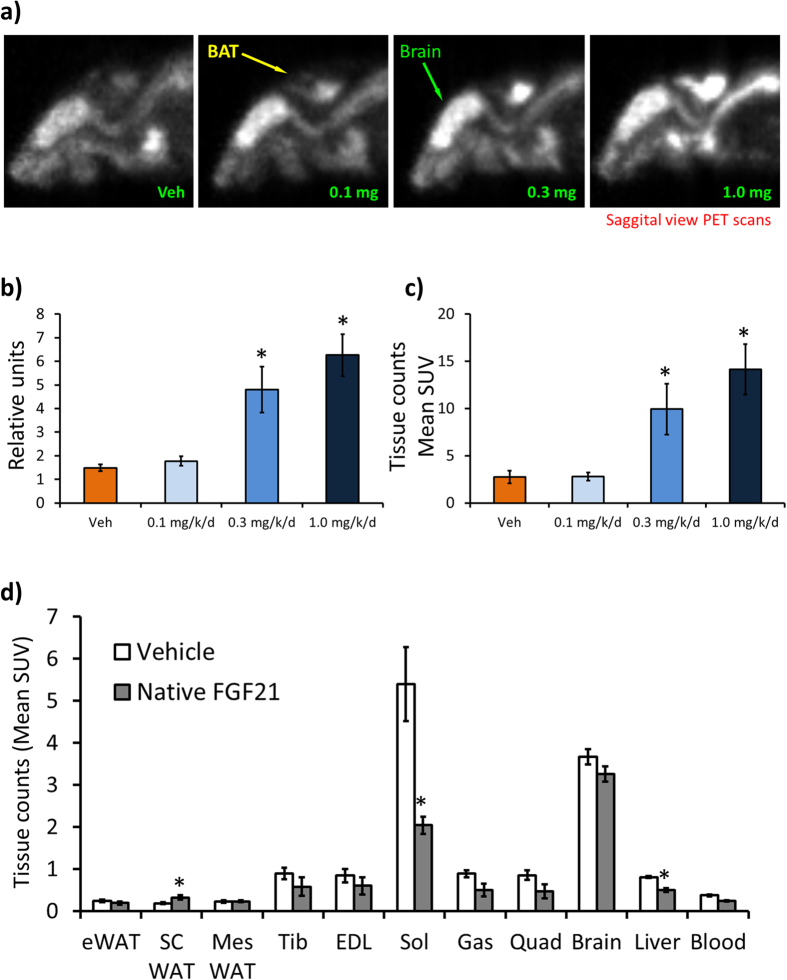
[^18^F]-FDG uptake in FGF21-treated DIO mice. **a**) Representative PET scan images in DIO mice administered native FGF21 at the indicated doses for two weeks. **b**) Region of interest (ROI) quantified from figure **a**) n = 6/group. **c**) [^18^F]-FDG counts from iBAT from DIOs administered native FGF21, n = 7–8/group. d) [^18^F]-FDG counts from indicated tissues from DIOs administered native FGF21 n = 7–8/group. Data represented as Mean ± SEM. * p < 0.05 by paired t-test.

Short-term cold exposure and β3-adrenergic stimulation causes a significant induction of FGF21 mRNA levels in BAT, without changing FGF21 plasma levels^23^ and previous work has demonstrated BAT is a target tissue of FGF21^24^. Next, we asked whether FGF21 administration in DIO mice modulates BAT activity and performed PET scans in DIO mice administered native FGF21 for two weeks. FGF21 caused a dose-dependent increase of [^18^F]-FDG uptake in iBAT compared to control (Fig. 3a). We quantitated the signal intensity which was significantly higher with the 0.3 mg/kg and 1.0 mg/kg treatment compared to control (Fig. 3b). We excised iBAT after PET imaging and counted the tissue for [^18^F]-FDG and consistent with tissue fluorescence, [^18^F]-FDG counts were significantly higher in the 0.3 mg/kg and 1.0 mg/kg treated animals compared to control (Fig. 3c). In addition, we excised and obtained [^18^F]-FDG counts for insulin responsive tissues (Fig. 3d). As expected, FGF21 significantly increased [^18^F]-FDG uptake in scWAT, however, the magnitude of uptake was less than that of [^18^F]-FDG uptake in iBAT (Fig. 3c). Consistent with no changes in GDR under clamp conditions (data not shown), there was no effect of FGF21 administration on [^18^F]-FDG uptake in tibialis, EDL, quad and brain (Fig. 3d), however, there was a significant decrease of [^18^F]-FDG uptake in the soleus and liver (Fig. 3d).

### BAT is redundant for metabolic effects of FGF21

**Figure 4:**
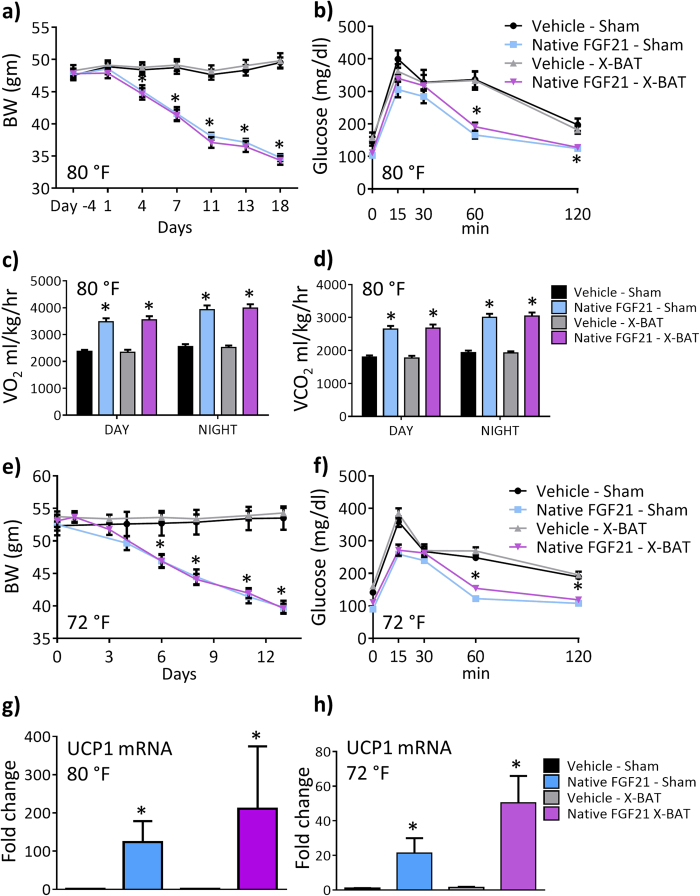
Role of interscapular BAT in FGF21 pharmacology. iBAT was excised (X-BAT), or sham surgery was performed in DIO mice and administered 0.85 mg/kg FGF21 or vehicle continuously via an osmotic minipump for 14 days. At 80 °F which is close to the thermoneutral zone in mice, **a**) BW, **b**) OGTT, **c**) oxygen consumption, **d**) CO_2_ production. The same experiment was performed in mice housed at 72 °F. **e**) BW, **f**) OGTT. UCP1 mRNA from inguinal white adipose tissue normalized to cyclin B as housekeeping gene from mice housed at **g**) 80 °F and **h**) 72 °F. n = 7–8 animals/group. Data represented as Mean ± SEM. *p < 0.05 by one way Anova and Dunnett’s posthoc test.

Since FGF21 increased [^18^F]-FDG uptake in DIO mice and FGF21 increases EE, we wanted to determine the contribution of BAT in this process. We excised either iBAT (X-BAT) or performed sham surgery in DIO mice that were housed under thermoneutral (80 °F) conditions, followed by continuous administration of either vehicle or native FGF21 for two weeks via a subcutaneously implanted minipump. As expected, FGF21 decreased BW in sham animals but interestingly, FGF21 decreased BW in X-BAT animals equally as observed in sham mice, compared to vehicle in sham or X-BAT mice (Fig. 4a). Glucose excursion during OGTT was decreased equally in sham, or X-BAT animals administered FGF21 (Fig. 4b). To detect potential changes in EE, we used CLAMS chambers to monitor metabolic rate of these animals. Consistent with previous reports, FGF21 treatment increased oxygen consumption (Fig. 4c) and CO_2_ production (Fig. 4d) however, these changes were similar in sham and X-BAT animals compared to control. FGF21 increased O_2_ consumption and CO_2_ production in sham, or X-BAT at night, compared to day, whereas there was no difference in animals administered vehicle (Fig. 4c,d). There was no change in ambulatory activity in FGF21 animals compared to control in sham and X-BAT mice (Supplementary Fig. S1b). Since mice are typically housed at ambient temperature (72 °F), we asked whether excision of iBAT would have any impact on the metabolic endpoints upon FGF21 treatment. Consistent with observations in sham and X-BAT animals housed at 80 °F, FGF21 decreased BW (Fig. 4e) and improved glucose excursion during OGTT (Fig. 4f) equally in sham, or X-BAT treated animals housed at 72 °F.

We performed gene expression analyses from inguinal white adipose tissue in sham and X-BAT animals housed at 80 °F (Fig. 4g) or 72 °F (Fig. 4h) treated with FGF21 or vehicle for 14 days. FGF21 increased the mRNA abundance of UCP1 in both sham and X-BAT animals in both temperature conditions. Increase of UCP1 in X-BAT animals was higher than sham animals at both temperatures, although they were not statistically different. X-BAT excision itself had no impact on any endpoints as evidenced by similar effects in each of the endpoints reported in this figure in sham and X-BAT animals administered vehicle. Western blots for UCP-1 showed that at both 80 °F and 72 °F, FGF21 administration in inguinal WAT of X-BAT animals were increased compared to all other groups (Supplementary Fig. S1c).

## Discussion

FGF21 has emerged as a promising therapeutic agent for metabolic diseases, since pharmacological administration of FGF21 causes improved glycemic control, lipid profile and weight loss in mice and rodents^25^. In non-human primates, FGF21 administration causes weight loss and improved lipid profile with moderate to no glycemic control^11^,^26^[Talukdar, S *et al.* Pfizer manuscript]. In humans, FGF21 administration decreases BW and improves lipid profile, without profound changes in glycemic endpoints, although Gaich *et al.*^7^ showed robust decrease of fasting insulin in the subjects. In rodents, body weight loss has been consistently reported in DIO models^18^,^22^, but when administered to ob/ob and db/db mice, there is little to no weight loss suggesting a contribution of the leptin pathway in this process^14^,^18^,^19^,^20^,^22^. The mechanism by which FGF21 causes weight loss also appears to be different in species, ranging from increased EE in DIO mice^4^,^6^,^18^, to decreased food intake in obese cynomolgus monkeys^26^ [Talukdar *et al.* Pfizer manuscript].

To interrogate FGF21 pharmacology in a different species and confirm dependence on the leptin pathway on metabolic endpoints such as BW, glycemic control, we used Zucker fatty rats Strain 185 Crl:ZUC-Lepr^fa^ 27 which lack the leptin receptor and develop obesity and hyperphagia. Consistent with observations made in ob/ob and db/db mice, native- or a long-acting FGF21 (PF-05231023) administration does not impact body weight or food intake, but improves glycemic control in Zucker rats. We performed clamp studies and demonstrated that improved glucose control is mediated by increased hepatic insulin responsiveness. Consistent with the fact that KLB is not expressed in skeletal muscle in mice^12^, there are no changes in muscle glucose disposal rate during clamp, establishing liver as a key organ integrating the beneficial actions of FGF21. Detailed mechanistic studies in livers from these rats demonstrated decreased lipogenesis, increased fatty acid oxidation and improved acute insulin signaling upon FGF21 treatment. To our knowledge, this is the first report demonstrating the pharmacology and mechanism of FGF21 action in this species. Importantly, these data demonstrate that glycemic control upon FGF21 administration is independent of BW suggesting the contribution of two independent pathways driving the process.

To further understand the role of pharmacological FGF21 on hepatic metabolism, we performed targeted metabolomics and lipidomics following two weeks of FGF21 treatment in DIO mice. Consistent with previous reports, there was a substantial effect of FGF21 on fatty acid metabolism as demonstrated by the significant reductions in many of the hepatic long chain fatty acid (LCFA) species. These data support and confirm the robust triglyceride lowering observed in preclinical species^25^ and humans^7^, that is further substantiated by the significant reductions in mature SREBP1 and the inhibition of acetate conversion into lipid. The increases in carnitine, acetyl-carnitine, and ketone bodies would support the increased oxidation of ^14^C-palmitate in the *ex vivo* liver homogenates. The 70% decrease in G-3-P could also contribute towards limiting TG synthesis. Taken together, in DIO mice FGF21 reduces hepatic and plasma lipids by promoting hepatic fatty acid oxidation and diminishing *de novo* lipogenesis capacity independent of the effects on weight loss and lipolysis.

Interestingly, pharmacological administration of FGF21 had dramatic effects of decreasing the concentrations of bile acids such as cholate and cholate-conjugated derivatives. FGF21 is directly regulated by FXR through an FXR/retinoid X receptor binding site in the 5′-flanking region on the FGF21 gene^28^ and FGF21 treatment decreases Cyp7a1 expression in mice^19^, although in other models there is no change^6^. One potential explanation could be that serum FGF19 levels are elevated upon chronic FGF21 treatment, which could account for the observed changes in BAs. However, FGF19 was not measured in this study although there is a reciprocal relationship between endogenous FGF19 and FGF21 in humans^29^. Moreover, hepatic amino acid concentrations were increased in mice treated with FGF21, which in turn could be due to the effects of FGF21 on gluconeogenesis in pre-clinical species as demonstrated by the improvement in clamp hepatic glucose production. Similarly, the effect of FGF21 reducing glucagon secretion might partially explain the increases in amino acids. Consequently, glucagon antagonism causes significant increases in amino acid concentration similar to what was observed in the mice treated with FGF21 for two-weeks^30^.

FGF21 promotes browning of white adipocytes^17^ and also works directly on brown cells^24^. In mice, FGF21 treatment decreases body weight and increases food intake when the data is expressed per unit body weight^4^,^18^. Consequently, FGF21 increases energy expenditure in these animals^4^, in addition to increasing beige/brown like markers in both white- and brown adipose tissue^17^. These findings of increased energy expenditure and beige/brown markers in white adipose tissue do not translate in higher species such as non-human primates where weight loss is driven almost exclusively by decrease in food intake^26^ [Talukdar, S. *et al.* manuscript] and food intake data is lacking in humans.

To determine the contribution of interscapular brown adipose tissue (iBAT) towards FGF21 pharmacology, we administered FGF21 and performed PET imaging in DIO mice. FGF21 caused a dose-dependent increase of [^18^F]-FDG uptake in iBAT of DIO mice housed at thermoneutrality (80 °F), suggesting improved BAT activity. We excised the iBAT and insulin responsive tissues from these animals and measured [^18^F]-FDG counts. Consistent with the PET data, FGF21 caused a dose dependent and significant increase of [^18^F]-FDG counts in iBAT and consistent with earlier reports, FGF21 increased glucose uptake in scWAT in these animals although the magnitude of glucose update was less than observed in iBAT. There was no change of [^18^F]-FDG uptake in skeletal muscle, although FGF21 decreased [^18^F]-FDG uptake in the gastrocnemius and liver.

Since FGF21 caused a dose-dependent increase of [^18^F]-FDG in iBAT suggesting improved BAT activity, to conclusively determine the contribution of BAT in FGF21 pharmacology at 80 °F which is close to thermoneutrality to eliminate potential confounders such as “primed” BAT at ambient temperature^31^, we excised iBAT and performed sham surgery in DIOs and administered vehicle or FGF21. All metabolic effects of FGF21 administration such as weight loss, improved glucose excursion during OGTT, O_2_ consumption and CO_2_ production were retained equally in sham and X-BAT animals, demonstrating the redundant role of iBAT in FGF21 pharmacology.

A previous study conducted iBAT excision in mice housed at ambient temperature (73.4 °F) and administered FGF21 to conclude iBAT is not required for FGF21 action^21^. However, in this report^21^, additional controls such as sham-surgery animals treated with vehicle or FGF21 were not run in parallel to the X-BAT animals to assert the conclusions. To robustly demonstrate the contribution of iBAT towards FGF21 pharmacology, we excised iBAT and conducted sham surgery in parallel and administered either vehicle or FGF21 at 72 °F. Similar to observations at 80 °F, all metabolic effects of FGF21 were retained in X-BAT and sham mice compared to control. The inclusion of sham animals administered vehicle or FGF21 in parallel with X-BAT animals allows us to conclude that iBAT does not play a role in FGF21 pharmacology on metabolic endpoints. BAT transplantation in recipient mice decreases insulin resistance and improves glucose tolerance in a dose-dependent manner^32^, suggesting the importance of BAT in rodent glucose homeostasis.

There could be several reasons why our result demonstrates a redundant role of BAT in the context of FGF21 biology in DIO mice. 1) The central nervous system (CNS) plays an important role in weight loss in FGF21-treated animals^16^, 2) it is possible that a longer dosing regimen could have brought out subtle differences, 3) the amount of glucose taken up by BAT upon FGF21 administration is a small fraction of circulating glucose^33^, and 4) our model eliminates only the iBAT and it is possible that other BAT depots^34^ or browning/beige-ing^31^ of white cells likely contribute to glucose uptake. It is also possible even after complete excision of iBAT, a significant portion of the classic BAT depot^35^ is retained, which contributes to mediating the effects of FGF21 treatment on body weight and glucose control.

It is important to emphasize that white adipose tissue plays an important role in FGF21 biology. For example, adipose KLB is required for acute insulin sensitizing effects of FGF21^8^, adipose FGFR1 KO^9^ and lipodystrophic animals^36^ are completely refractory to the effects of pharmacological FGF21 administration such as weight loss and glucose tolerance, however, in the lipodystrophic animals, liver remained sensitive to the effects of FGF21^36^. Further evidence has demonstrated that adiponectin mediates the glycemic and insulin sensitizing effects of FGF21^15^ and adiponectin has been proposed to act on the liver and mediate systemic effects of FGF21^37^. Our data is consistent with a previous study^17^ demonstrating FGF21 increases the “browning” program in white fat as shown by increased UCP-1 mRNA expression. The authors^17^ speculated that thermogenic effects of FGF21 could account for the beneficial role of FGF21 *in vivo*^17^. Our data demonstrates in spite of BAT activation and browning of the inguinal white adipose tissue upon FGF21 administration, these processes are not major contributors to weight loss and glycemic control in DIO mice after two weeks of treatment.

Taken together, this is the first report demonstrating metabolic effects of FGF21 in Zucker fatty rats. There are several important aspects of this manuscript. First, we demonstrate FGF21 pharmacology and mechanism of action in an obese rat model that is impaired in the leptin signaling pathway. Consistent with observations in ob/ob and db/db mice, FGF21 treatment does not cause weight loss, demonstrating the leptin-pathway dependence for weight loss in another species. Second, improved glycemic control in Zuckers despite lack of weight loss suggests a direct, leptin pathway independent effect of FGF21 on glucose control. We demonstrate that the liver integrates the actions of FGF21 resulting in improvement of glucose tolerance in rodents. Third, harnessing the BAT for improved metabolic endpoints has been pursued as a potential therapeutic approach. In the context of FGF21, which acts on the BAT^17^, we demonstrate iBAT is redundant, despite improved activity, as demonstrated by a dose dependent increase of [^18^F]-FDG glucose. This observation is not surprising, because FGF21-mediated weight loss in rodents is mediated by the CNS^16^ and glycemic control is likely driven by the periphery where white adipose tissue plays an important role^15^ along with the liver^4^. Finally, we provide data to justify continued investment into FGF21 pharmacology as the next generation therapeutic in the metabolic disease area.

## Methods

### OGTT and hyperinsulinemic-euglycemic clamp in Zucker LepRfa rats

OGTT was conducted as described^38^. On day 15, after 6 h fasting, rats were orally gavaged (1 g/kg BW) with dextrose (Hospira, Inc) and blood glucose was measured from tail vein using OneTouch Ultra (LifeScan, Inc) at indicated times. Clamps were performed as described^39^. Briefly, five days before clamp experiments, animals were chronically cannulated in the jugular vein under single-dose anesthesia. For blood sampling, one carotid cannula was implanted and all cannulae were tunneled subcutaneously, exteriorized at the back of the neck, and were encased in an infusion harness. The day prior to clamp studies, animals were fasted overnight. Throughout the clamp animals were unrestrained in their cages and had free access to water. At −60 min a priming dose of 7.5 μCi of D-[3-3H] glucose was administered into the carotid, and a constant-rate (0.167 μCi/min; 16.7 μL/min) infusion of D-[3-3H] glucose was started at –70 min. Blood samples were collected at −10 min and 0 min for measurement of tracer specific activity and basal HGP. At 0 min, a variable infusion of glucose (50% dextrose; Hospira, Inc) and tracer-plus-insulin (16 mU/kg/min; Humulin® R; Eli Lilly and Company) infusions were started simultaneously. Blood was sampled from the carotid artery at 10-min intervals and immediately analyzed for glucose. Glucose infusion was adjusted to maintain blood glucose at 100 mg/dL. Finally, blood samples were collected at 110 min and 120 min for measurement of tracer specific activity. All experiments involving animals were carried out per established guidelines and protocols that were reviewed and approved by the Institutional IACUC.

### PET scans

20 male C57Bl6/J DIO mice aged 16 weeks, were administered vehicle or FGF21 at 0.1, 0.3, and 1 mg/kg/day continuously via surgically implanted mini-pump (Alzet® mini-pump (Model #2002, Durect Corporation) for 8 days. Mice were fasted overnight and [^18^F]-FDG PET was performed using a Siemens Focus 220 microPET at 60 minutes post [^18^F]-FDG injection (~47 MBq, iv). To minimize background BAT FDG uptake, animals were maintained at 29.5 ± 2 °C beginning at 18 hours prior to scanning and up until time of scan. During scanning, body temperature was maintained using a heat lamp. After scanning was completed, animals were immediately sacrificed and BAT was dissected out and counted to determine the FDG uptake per gram of tissue using a Wallac Wizard 1470 gamma counter. Regions of interest (ROIs) were drawn around BAT images to determine mean and max [^18^F]-FDG uptake then analyzed using Analyze Software (version 7.0, developed by Mayo Clinic). ROI-derived and tissue counting data were compared for correlation.

### BAT studies

32, 21 week old, male C57BL6/J DIO mice were obtained from the Jackson Laboratories (Bar Harbor, ME), fed 60% high fat diet (Research Diets D12492). Mice were acclimatized for 2 weeks at 72 °F (ambient) then moved to 80 °F that is close to thermoneutral zone, to acclimate another week prior to baseline randomization and subsequent surgery. For experiments at ambient temperature, mice were housed at 72 °F throughout the study. Animals were allocated into 4 groups of n = 8 based on BW and glucose levels to achieve equal distribution of these parameters in each group. On day 0, all animals underwent surgery for the implantation of an Alzet® mini-pump (Model #2002, Durect Corporation) for continuous delivery of either vehicle (PBS) or 0.85 mg/kg/day native FGF21. The iBAT was surgically removed at the time of mini-pump implant in those mice allocated into the X-BAT groups. VO_2_ and VCO_2_ were measured for 24 hours of fed and 24 hours of fasted conditions using a comprehensive laboratory animal monitoring system (CLAMS) equipped with an Oxymax Open Circuit Calorimeter (Columbus Instruments, Columbus, OH) as previously described (n = 8/group)^20^,^30^. RT-PCR One-Step (Qiagen) was used to determine the expression of UCP-1 by standard PCR protocol.

### Acute insulin response

Acute insulin response was conducted as described^40^. Briefly, rats were fasted overnight and 2 hours prior to the procedure, were administered a SC dose of 1.32 mg/kg FGF21. Under anesthesia, 0.35 U kg^−1^ insulin was injected via inferior vena cava. Basal liver, muscle and adipose biopsies were obtained prior to insulin administration. Livers and muscle biopsies were obtained after 3 min and adipose biopsies were obtained after 5 min of insulin administration.

### Western blots

Western blots for P-Akt and total Akt were performed as described^40^. For UCP1 and tubulin, inguinal white adipose tissue was lysed using 100 μl of RIPA buffer containing protease inhibitors (phosphatase inhibitor cocktail #3 (Sigma cat# P0044) 10 ul/1ml, and PMSF Cell Signaling (cat#8553S)) at 100 nM final concentration at 4 °C. 20 μg of inguinal fat lysates were loaded and 5 ug of lean mouse BAT lysate was used as positive control. UCP1 antibody (5901) at 1:10000 in SuperBlock in TBS Thermoscientific overnight at 4 °C. Detection was done by using SuperSignal WestDura , Thermo scientific (cat#34076). Tubulin was used as loading control at 1:10000 in SuperBlock in TBS Thermoscientific overnight at 4 °C and 1:10000 goat @rabbit-HRP (Cell Signaling) at 1:10000 SuperBlock in TBS Thermoscientific for 1 hr at room temperature.

### Metabolomic analysis

DIO mice were administered 10 mg/kg PF-05231023 twice a week for 2 weeks. Mice were fasted overnight, sacrificed and livers were excised and stored at −80 °C. Livers were sent to Metabolon (Metabolon Inc, Durham, NC) for metabolomic analysis using their proprietary methods.

### Statistical Analysis

Significant differences between groups for dependent variables were tested using either single-factor (group) analysis of variance (ANOVA) or two-way (group and time) ANOVA. For single-factor and two-way ANOVAs, Tukey’s multiple comparisons test was used for post hoc analyses for between-group comparisons. To assess differences between the treated and untreated groups, the ratio in the average delta CT values were compared. Analyses were conducted using GraphPad Prism Statistical Software Version 6.0 (San Diego, CA).

## Additional Information

**How to cite this article**: Bernardo, B. *et al.* FGF21 does not require interscapular brown adipose tissue and improves liver metabolic profile in animal models of obesity and insulin-resistance. *Sci. Rep.* **5**, 11382; doi: 10.1038/srep11382 (2015).

## Supplementary Material

Supplementary Information

## Figures and Tables

**Table 1:** Heat map of liver LCFA profiles showing relative fold change of the metabolites on day 14 between PF-05231023 vs. vehicle treated samples.

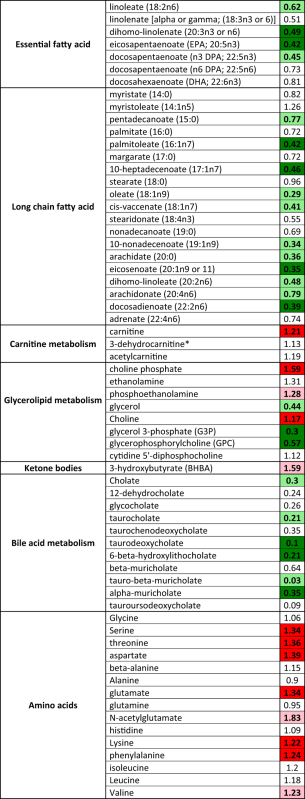

Statistically significant reductions are highlighted in (dark green and red indicates p < 0.05, light green and red indicates 0.05 < p < 0.1) using paired t-test. No color indicates lack of significant change.
