# Supplementary material for: FGF21 does not require interscapular brown adipose tissue and improves liver metabolic profile in animal models of obesity and insulin-resistance
Source: Sci Rep. 2015 Jul 8;5:11382. doi: 10.1038/srep11382 (PMC4495598; doi:10.1038/srep11382)

FGF21 does not require interscapular brown adipose tissue and improves liver metabolic profile in animal models of obesity and insulin-resistance

Barbara Bernardo^1^, Min Lu^2,3^, Gautam Bandyopadhyay^2^, Pingping Li^2^, Yingjiang Zhou^1^, Jie Huang^3^, Nancy Levin^3^, Eva M. Tomas^1^, Roberto A. Calle^1^, Derek M. Erion^1^, Timothy P. Rolph^1^, Martin Brenner^1^, Saswata Talukdar^1,^*

1, Cardiovascular Metabolic and Endocrine Diseases (CVMED) Pfizer, Inc. 610 Main Street, Cambridge, MA 02139, USA.

2, Department of Medicine, University of California, San Diego, 9500 Gilman Drive, La Jolla, CA 92093, USA.

3, CovX Research, Pfizer WRD, USA.

* Corresponding author, [Saswata.Talukdar@pfizer.com](mailto:Saswata.Talukdar@pfizer.com)

**Supplementary Figure S1.**

a) Eight week old Zucker rats were administered vehicle, 1.32 mg/kg/day native FGF21, or 3 mg/kg and 10 mg/kg PF-05231023 subcutaneously twice a week for two weeks. Clamp glucose disposal rate (GDR) in these animals, n = 6 animals/group. b) iBAT was excised (X-BAT), or sham surgery was performed in DIO mice and administered 0.85 mg/kg FGF21 or vehicle continuously via an osmotic minipump for 14 days. Ambulatory activity during and night in sham and X-BAT animals administered vehicle or FGF21. n = 7 - 8 animals/group. c) Inguinal white adipose tissue was excised from sham and X-BAT animals administered vehicle or FGF21 and western blot analyses was performed and stained for UCP1 and tubulin as house-keeping gene. UCP1 and tubulin western blots were run on independent gels in the order shown in the figure. BAT lysate from lean mice were used as positive control. Blots are shown as they were run. n = 4 – 6 animals/group. Data represented as Mean ± SEM.


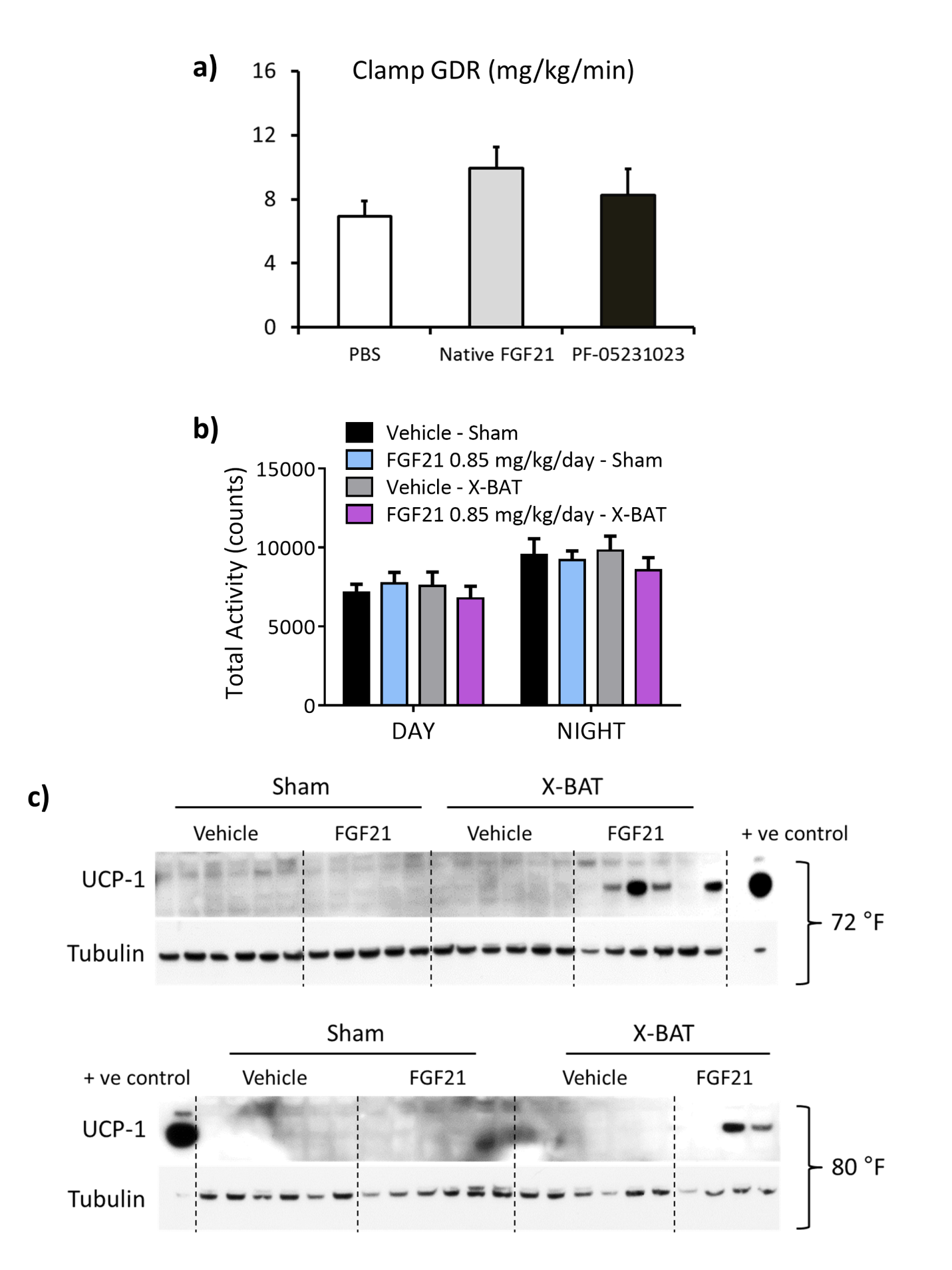

Supplement: Supplementary Information [file srep11382-s1.docx]
